# Supplementary material for: Traces of electron-phonon coupling in one-dimensional cuprates
Source: Nat Commun. 2023 May 30;14:3129. doi: 10.1038/s41467-023-38408-6 (PMC10229634; doi:10.1038/s41467-023-38408-6)
Supplement: Supplementary file 1 — Supplementary Information [file 41467_2023_38408_MOESM1_ESM.pdf]

Supplementary Information
Traces of Electron-Phonon Coupling in One-Dimensional Cuprates

Ta Tang,[1,2] Brian Moritz,[2] Cheng Peng,[2] Zhi-Xun Shen,[1,2,3,4] and Thomas P. Devereaux[2,4,5,*]

[1]*Department of Applied Physics, Stanford University, California 94305, USA.*

[2]*Stanford Institute for Materials and Energy Sciences, SLAC National Accelerator Laboratory, 2575 Sand Hill Road, Menlo Park, California 94025, USA.*

[3]*Department of Physics, Stanford University, Stanford CA 94305, USA.*

[4]*Geballe Laboratory for Advanced Materials, Stanford University, Stanford, CA 94305, USA.*

[5]*Department of Materials Science and Engineering, Stanford University, Stanford CA 94305, USA.*

(Dated: April 14, 2023)

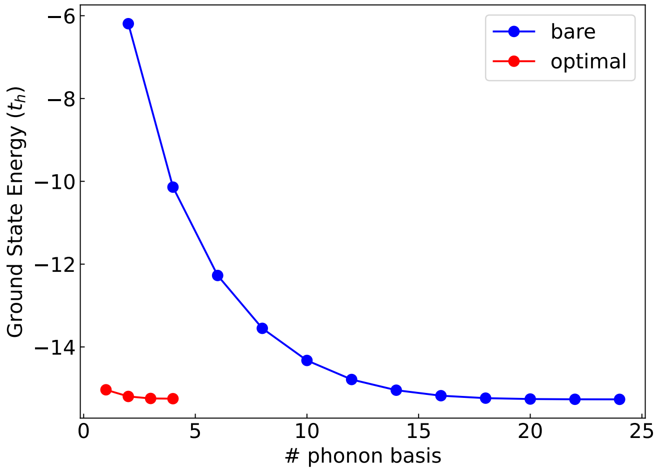

FIG. S1. **Ground state energy convergence of LBO on an 8-site chain at half filling.** The blue curve corresponds to using bare phonon basis without LBO and we need about 20 bare phonon basis to converge the ground state. The red curve is LBO with 20 bare phonon basis and the ground state is well converged with just 3 optimal phonon basis.

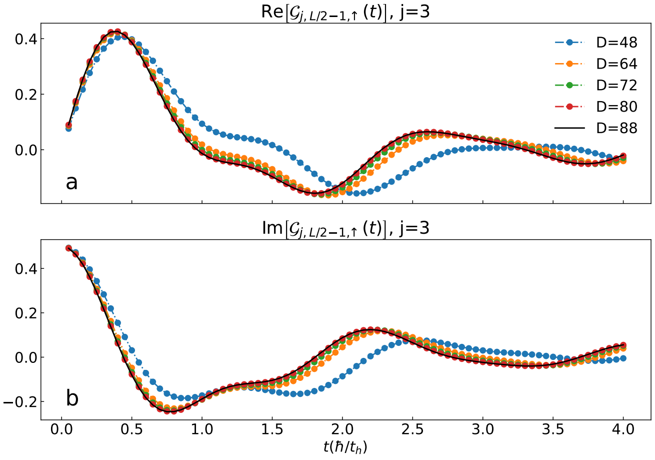

FIG. S2. **Time evolution convergence with respect to D.** Time evolution convergence of the lesser Green's function with respect to the number of bare local basis dimension D on an 8-site chain at half filling without dynamical LBO. The time step is $\delta t = 0.05$ here. The lesser Green's function is well converged with $D = 80$ (20 bare phonon basis).

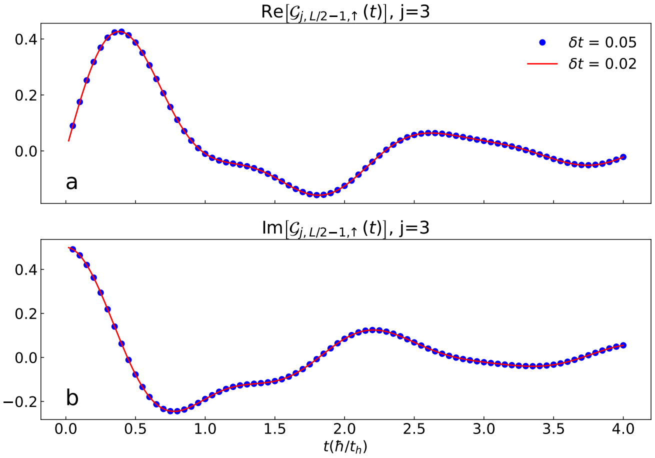

FIG. S3. **Time evolution convergence with respect to time step.** Time evolution convergence of the lesser Green's function with respect to time step on an 8-site chain at half filling without dynamical LBO. We use $D = 80$ here and we see that time evolution with $\delta t = 0.05$ is well converged.

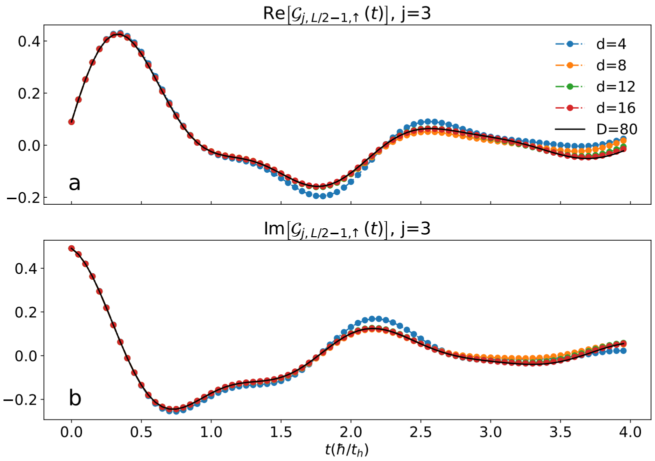

FIG. S4. **Time evolution convergence with respect to *d*.** Time evolution convergence of the lesser Green's function with respect to optimal basis number with dynamical LBO. We fix $D = 80$ and $\delta t = 0.05$ and we see $d = 12$ is enough for good convergence.

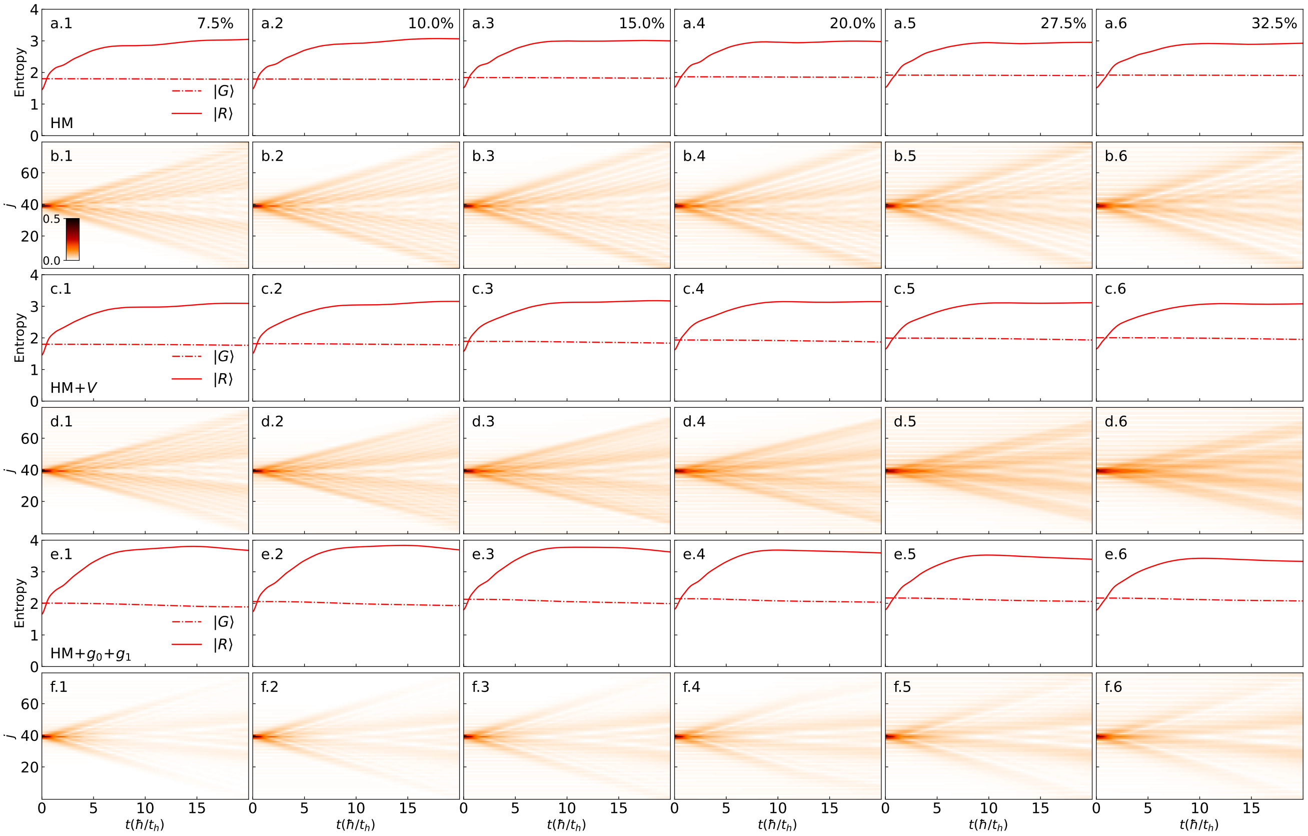

FIG. S5. **Entropy growth and lesser Green's function.** Raw data of entropy growth (A, C and E) during time evolution and lesser Green's function (B, D and F) of different models at different dopings obtained on an 80-site chain. The single particle spectral functions shown in Fig. 3 are obtained from these raw data. The convergence of entropy growth over time has been verified by comparing to results obtained with larger bond dimension m and larger bare phonon basis dimension D. For the Hubbard and extended Hubbard models, m=800 entropy values only differ slightly from the m=1200 results at late times (around 1-2% relative error at the last time step). For Hubbard-extended Holstein model, entropy values for m=900 and D=100 overlap with those for m=800 and D=80.

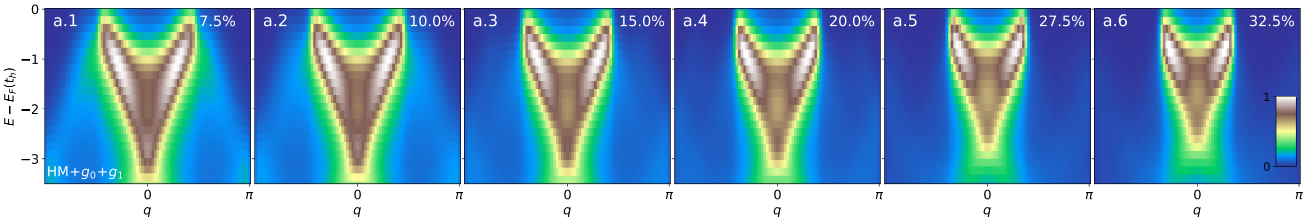

FIG. S6. **Spectral functions with larger broadening.** Single particle spectral function for the Hubbard-extended Holstein model with larger energy broadening: $0.3t_h$ Lorentzian broadening plus $0.2t_h$ additional Gaussian broadening, similar to the broadening used in Ref.[1].

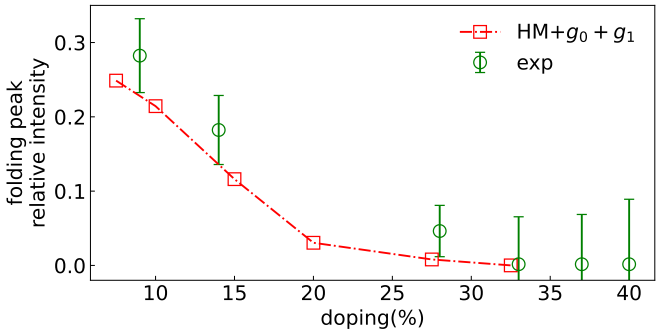

FIG. S7. **Holon folding peak intensity relative to the main holon peak as a function of doping.** The red square represents data from simulation of Hubbard-extended Holstein model. The intensity is extracted by fitting MDC cut with sum of Gaussian peaks. The MDC cut is chosen to be $\sim t_h$ above the holon bottom and is extracted from Fig. S6. The green circle represents experimental data from Ref.[1].

SUPPLEMENTARY REFERENCES

* tpd@stanford.edu

[1] Zhuoyu Chen, Yao Wang, Slavko N. Rebec, Tao Jia, Makoto Hashimoto, Donghui Lu, Brian Moritz, Robert G. Moore, Thomas P. Devereaux, and Zhi-Xun Shen. Anomalously strong near-neighbor attraction in doped 1D cuprate chains. *Science*, September 2021.
